# Exploring the impact of home-based Vojta therapy on gait performance in individuals with Down syndrome: a preliminary feasibility study

**DOI:** 10.3389/fneur.2025.1537635

**Published:** 2025-03-11

**Authors:** Guoping Qian, Ewelina Perzanowska, Dominika Wilczyńska, Mirela Kozakiewicz, Hongli Yu, Marcelina Hejła, Zbigniew Ossowski

**Affiliations:** ^1^Department of Physical Culture, Gdansk University of Physical Education and Sport, Gdansk, Poland; ^2^Faculty of Social and Humanities, WSB Merito University Gdansk, Gdańsk, Poland; ^3^College of Physical Education, Sichuan University of Science & Engineering, Zigong, Sichuan, China

**Keywords:** home-based Vojta therapy, spatiotemporal gait parameters, Down syndrome, VICON, feasibility study

## Abstract

**Background:**

Vojta therapy (VT) enhances postural control and improves gait abilities. However, there is limited evidence regarding the impact of home-based VT on individuals with Down syndrome (DS).

**Objective:**

This study aimed to assess the feasibility and preliminary effects of a two-week home-based VT program on spatiotemporal gait parameters in individuals with DS.

**Methods:**

Sixteen individuals with DS (mean age = 17.88 ± 4.57 years, 8 females) participated in a two-week home-based VT program. Feasibility was measured through adherence rates and the occurrence of adverse events. Spatiotemporal gait parameters were evaluated before and after the intervention using the Vicon motion capture system.

**Results:**

All participants (100%) successfully completed the home-based VT program with no reported adverse events. Significant improvements were observed in walking speed, cadence, step time (left and right), stride time (left and right), step length (left and right), stride length (left and right), and single support (left and right) (*p* < 0.05).

**Conclusion:**

This preliminary study suggests that home-based VT is a feasible approach and can lead to meaningful improvements in spatiotemporal gait parameters for individuals with DS. Further research with larger sample sizes, more robust designs, and extended follow-up periods is recommended.

## 1 Introduction

Down syndrome (DS, OMIM #190685) or trisomy 21 is caused by the triplication of the whole or portion of chromosome 21 (1). It is the most prevalent genetic neurological disorder with intellectual disabilities and movement impairment, occurring in about 1 in 800 births globally (2). DS manifests clinically a variety of symptoms that include muscle hypotonia, short stature, atlantoaxial instability, intellectual disability, and congenital heart defects (2–4). Additionally, DS patients carry an increased risk of various medical conditions such as respiratory disease, gastrointestinal malformations, osteoporosis, thyroid dysfunction, epilepsy, and metabolic disorders (3, 5–8).

Musculoskeletal alteration of DS manifests as motor and coordination problems most frequently leading to abnormal gait patterns (9). Gait abnormalities often observed in DS individuals are reduced walk speeds, reduced stride length, increased stride width, increased hip flexion (10). Other typical characteristics include hip external rotation, increased knee flexion and valgus, and tibial external rotation (11). All these issues are mainly due to hypotonia, joint stiffness, and ligamentous laxity that disrupt intermuscular coordination and proprioceptive processing to ultimately impairing postural control and gait (12). These gait abnormalities continue into childhood, with studies demonstrating that DS children reach motor milestones, including independent walking, much later than typically developing children—often between 24 and 36 months—compared to the usual 11 to 15 months for typically developing children (9, 13–15).

These motor dysfunctions limit physical activity and quality of life, affecting daily living activities and increasing reliance on others (16). Consequently, rehabilitation programs, therapeutic exercises, and physiotherapy are essential for promoting independence and mobility in persons with DS. For example, studies have shown that massage treatment can significantly improve motor function in children with DS (17, 18), and whole-body vibration training has been shown to enhance balance after 20 weeks (19). Additionally, various reviews have confirmed the effectiveness of physiotherapy in improving the overall quality of life and aiding rehabilitation in people with DS (20, 21).

Vojta therapy (VT) is a widely used physiotherapy technique developed by Vaclav Vojta (22). This neuromodulative treatment based on reflex locomotion principles and aims to promote typical innate patterns, and improve postural control. VT is established on the theory of postural and gait control pattern generators, whereby distinct areas of the body are stimulated to provoke corrective movements. The “bottom-up” concept is believed to take advantage of neuroplasticity (23–25) to enable patient recovery. Experiments have determined VT effectiveness in enhancing motor function in healthy adults and cerebral palsy (CP) patients as measured by functional near-infrared spectroscopy or surface electromyography (26, 27).Therefore, routine application of VT in the treatment of neuromotor pathologies such as CP, multiple sclerosis (MS), and hypotonia, has revealed gait, sitting balance, balance, and gross motor function improvement (28–32). VT has also been effective in improving postural control in stroke patients (33), and a systematic review of 10 trials involving 522 subjects showed its impressive benefits in the management of respiratory distress syndrome in preterm infants (34).

Home-based physiotherapy has gained much popularity over the last few years due to its convenience and cost-effectiveness. By allowing patients to receive treatment in their own homes (35), this approach promotes greater independence compared to traditional in-clinic physiotherapy (36). Numerous studies suggest that home-based physiotherapy is effective in enhancing mobility and improving daily living activities (37–39). Although VT and home-based physiotherapy have both been demonstrated to have beneficial outcomes, feasibility and efficacy of home-based VT in persons with DS, and more specifically the effect on spatiotemporal gait parameter improvement, is a research gap. Since the study was of an exploratory nature, a pre-post intervention study design was undertaken to investigate the feasibility and initial effect of home-based VT on the improvement of spatiotemporal gait parameters in persons with DS. This study enables evaluation of within-subject change before and after home-based VT, which is highly applicable to research on novel rehabilitation interventions in a population where comparatively little research has been conducted. The aim of this research, therefore, was to determine the feasibility and preliminary effect of a home-based VT intervention on spatiotemporal gait parameters in adults with DS with preliminary evidence for the design of larger, controlled studies.

## 2 Methods and materials

### 2.1 Research design

The study was a single-arm, prospective preliminary feasibility trial, employing a pre-post intervention design to evaluate the feasibility and effects of home-based VT on spatiotemporal gait parameters in individuals with DS. The study strictly adhered to the Transparent Reporting of Evaluations with Nonrandomised Designs (TREND) guidelines to ensure rigorous reporting standards (40).

### 2.2 Participants

Participants were recruited from different rehabilitation centers in Gdansk, Poland between April and May 2023. The inclusion criteria were: (1) individuals aged 10 to 30 years; (2) interest in participating in the home-based VT program; (3) the ability to follow instructions, with parental support to cooperate with the home-based VT. Exclusion criteria included: (1) participation in a structured physiotherapy program within 3 months prior to the study; (2) previous abdominal or head surgeries (due to risk associated with reflex creeping during treatment); (3) presence of inflammatory disease or acute fever; and (4) any pharmacological or surgical treatment affecting the nervous system.

### 2.3 Ethics approval

Ethical approval was granted by the Bioethics Commission of the District Medical Chamber in Gdansk (KB/23–23). All procedures were carried out in compliance with the ethical standards established in the Declaration of Helsinki (1975) and its latest amendments. The study procedures, benefits, and potential risks were thoroughly explained to participants and their families. Written informed consent was acquired from all participants or their legal guardians if participants were unable to provide consent independently.

### 2.4 Intervention

Participants underwent a two-week home-based VT program as the only intervention during the study, with the recommended frequency being once per day, ideally in the afternoon. Each session consisted of 2-min exercises on each side in the reflex creeping position (2 min × 2 times for both left and right sides), with a 2–3-min break between positions, totaling approximately 20 min per session. No additional physical therapy or exercises conducted before, during, or after the VT sessions. This ensures that the observed effects on gait parameters can be directly attributed to VT. According to VT recommendations, session duration typically ranges from 5 to 20 min per session (22, 41). Previous studies have demonstrated that a single 20-min VT session per day can effectively improve shoulder function in individuals with subacromial impingement syndrome (42), while being a feasible duration that helps prevent excessive fatigue, supporting the feasibility and effectiveness of our chosen intervention duration. In addition, given the home-based setting, a single daily session was chosen to balance efficacy and adherence while maintaining alignment with VT principles. A previous study emphasizes that session frequency must balance effectiveness with feasibility, as excessive sessions may reduce compliance (43). This study adopted a practical intervention duration and frequency suitable for home-based settings.

An initial fconsultation in person with an experienced physiotherapist carried out to illustrate the VT techniques, with comprehensive guidance to parents on how to use VT at home. Key points included maintaining symmetry during exercises, ensuring proper stabilization of support points, and positioning the head correctly. Parents practiced under direct supervision to ensure they could accurately apply VT at home. To enhance learning, Supplementary Material was provided, such as written information and video illustrations.

For maintaining quality and uniformity of home sessions, physiotherapist provided day-to-day supervision by video call with on-the-spot correction and feedback. Parents were also asked to videograph sessions for observation and modification whenever necessary. Systematic monitoring ensured adherence to protocol, reduced errors, and made adjustments in time based on individual response. VT sessions were conducted by the children in shorts only to allow visualization of muscle chain activity.

### 2.5 Outcomes

Spatiotemporal gait parameters, i.e., walking speed (m/s), cadence (steps/min), step time (sec), step length (m), stride time (sec), stride length (m), double support % of the gait cycle (% GC), and single support (% GC) were recorded at baseline and post-intervention. Baseline measurements were conducted 48 h before the home-based VT program to minimize the influence of physiological factors, such as fatigue or recent physical activity. Post-intervention gait data were recorded 48 h following the final home-based VT session to avoid interfering with the measurements by the acute reflex activation effects of VT.

Measurements were captured with a 10-camera Vicon motion capture system (VICON, Oxford Metrics Limited, UK), functioning at a frequency of 100 Hz. The Vicon system is recognized for its exceptional accuracy and precision (44), with a mean absolute error of 0.15 mm in static situations and under 2 mm during dynamic motions (44). The Vicon system captures marker positions during walking with an accuracy of −0.08 mm and an uncertainty of 0.33 mm (45). Sixteen reflective markers, each measuring 2.5 cm in diameter, were affixed to the left and right lower limbs and the spine. Joint centers were defined according to the plugin-gait model (46). The markers were positioned at the bilateral anterior superior iliac spines, posterior superior iliac spine, the middle and lower 1/3 of the femur, the lateral line of the knee joint, the lateral 1/3 of the tibia, lateral malleolus, calcaneus, and dorsal second metatarsophalangeal joint. Participants walked barefoot along a 10-meter path at a self-selected pace, and data from five repeated trials were collected.

Spatiotemporal gait data were processed using Vicon Nexus 2.9.3 software (Vicon, Oxford Metrics). Preliminary marker reconstruction and labeling followed standard Nexus procedures, and missing data were interpolated using the Woltring filter with default Vicon settings. Gait events, such as heel-strike (HS) and toe-off (TO), were automatically detected using Vicon Nexus 2.9.3 software via the Plug-in-Gait model, which applies kinematic-based detection algorithms. All gait events were manually reviewed and corrected when necessary to ensure accuracy. Spatiotemporal parameters were derived from detected gait events using standard temporal calculations within Vicon Nexus software. To minimize noise while preserving movement characteristics, raw marker trajectories were smoothed using a second-order zero-lag Butterworth low-pass filter. These measurements were performed at the Laboratory of Physical Effort and Genetics in Sport at the Gdansk University of Physical Education and Sport (GUPES) in Poland.

### 2.6 Feasibility

The feasibility of the two-week home-based VT program was evaluated based on three criteria: (1) participant adherence, described as the quantity of participants who completed the program; (2) safety, monitored through the reporting of any adverse events related to the therapy; and (3) preliminary effectiveness of the intervention (47).

### 2.7 Sample size calculation

Since this study was designed as a preliminary feasibility study, no formal power calculation was performed. The target sample size was set at 16 participants.

### 2.8 Data analysis

Descriptive statistics were employed to examine demographic characteristics and study outcomes, with continuous variables reported as mean ± standard deviation (S.D.) or median (interquartile range, IQR). Grubb’s test was used to identify outliers. The Shapiro–Wilk test was conducted to evaluate the normality of the differences between pre- and post-intervention spatiotemporal gait parameters. For normally distributed data, a paired-sample t-test was conducted to assess before and after intervention effects.

Data were analyzed using Microsoft Excel 2016 (Microsoft Corp.) and SPSS version 20.0 (IBM SPSS). Statistical significance was defined as *p* < 0.05 (two-tailed).

## 3 Results

### 3.1 Study population

Sixteen participants (*n* = 8 female, 50%; *n* = 8 male, 50%) with a mean age of 17.88 ± 4.57 years were assessed by an experienced physiotherapist and recruited for this study. Among the participants, hypotonia was observed in 13 individuals (81%), and visual impairment was present in 12 individuals (75%). Additionally, 1 participant (6%) had a history of respiratory failure. Baseline characteristics for all participants are presented in Table 1, with further details for each subgroup provided in Supplementary Table 1.

**Table 1:** Characteristics of the participants (*n* = 16).

Gender (F/M)	8/8
Age (year)	17.88 ± 4.57
Height (cm)	157.5 ± 11.5
Weight (kg)	61.41 ± 11.13
BMI (kg/m2)	24.88 ± 4.53
Ethnicity (white)	16 (100%)
respiratory failure	1 (6%)
Visual impairment	12 (75%)
Hypotonia	13 (81%)

Values are presented as a number or mean ± standard deviation (S.D.). BMI, body mass index; cm, centimeter; F, female; M, male; kg, kilograms; m, meter.

### 3.2 Outcomes

The Shapiro–Wilk test indicated that most variables followed a normal distribution. These variables—walking speed, cadence, step length (left and right), stride length (left and right), stride time (left and right), step time (left and right), single support (left), and double support—were examined with a paired samples t-test. Single support (right), which did not follow a normal distribution, was evaluated using the Wilcoxon signed-rank test. Changes in spatiotemporal gait parameters after home-based VT are reported in Table 2.

**Table 2:** Change in spatiotemporal gait parameters after home-based vojta therapy (*n* = 16).

Variables	Pre-test (mean ± S.D./median)	Post-test (mean ± S.D./median)	*T*-value/*Z*-value	Hedge’s g/r-value
Spatiotemporal				
Walking speed (m/s)**	0.86 ± 0.24	1.17 ± 0.29	−4.73	1.12
Spatial				
Step length (left) (m)**	0.50 ± 0.11	0.59 ± 0.10	−3.75	0.89
Step length (right) (m)**	0.49 ± 0.10	0.59 ± 0.10	−4.33	1.02
Stride length (left) (m)**	0.98 ± 0.21	1.18 ± 0.19	−4.19	0.99
Stride length (right) (m)**	0.99 ± 0.21	1.15 ± 0.21	−3.17	0.75
Temporal				
Stride time (left) (s)**	1.17 ± 0.14	1.04 ± 0.16	3.09	0.73
Stride time (right) (s)**	1.21 ± 0.13	1.01 ± 0.13	5.95	1.41
Step time (left) (s)**	0.61 ± 0.08	0.53 ± 0.10	3.13	0.74
Step time (right) (s)*	0.58 ± 0.07	0.52 ± 0.07	2.79	0.66
Cadence (steps/min)**	102.57 ± 11.54	119.36 ± 14.39	−4.89	1.16
Temporophasic				
Single support (left) (% GC)*	0.46 ± 0.04	0.42 ± 0.05	2.82	0.67
Single support (right) (% GC)**	0.50 (0.50, 0.50)	0.42 (0.40, 0.40)	2.67	0.67
Double support (% GC)	0.24 ± 0.09	0.21 ± 0.07	1.57	0.37

Each treatment variable indicates significant differences at **p < 0.01 and *p < 0.05. SD, standard deviation; m/s, meters per second; m, meter; s, second; steps/min, steps per minute; % GC, percentage of the gait cycle.

### 3.3 Spatiotemporal parameters

**Figure 1:**
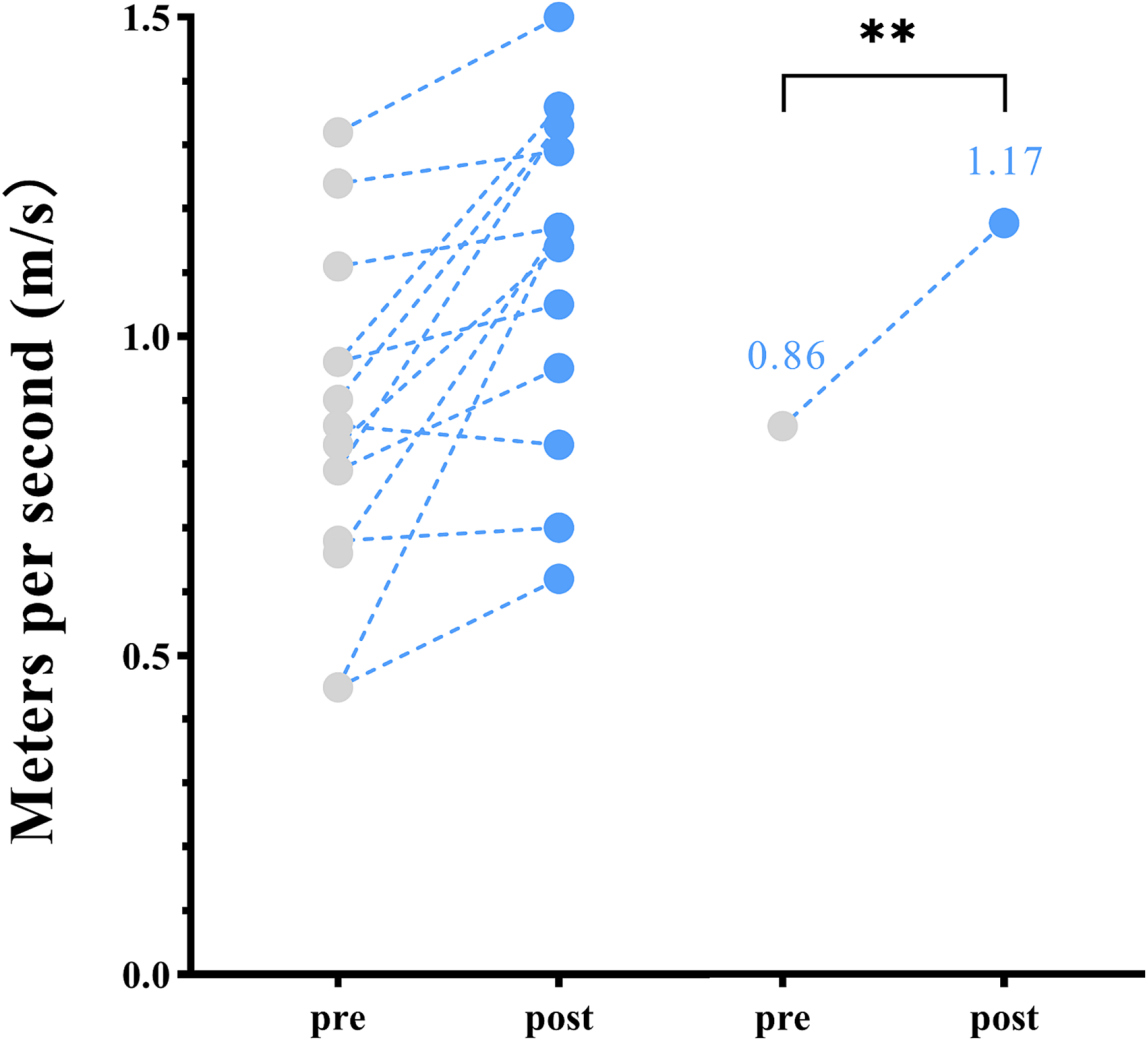
Spatiotemporal parameters—changes in walking speed between pre- and post-home-based vojta therapy (VT) program. The circles on the left represent individual participants; the circles on the right represent the group mean of pre- and post-intervention assessments.

Following 2 weeks of home-based VT, participants with DS demonstrated a significant increase in walking speed, from a pre-assessment mean ± SD of 0.86 ± 0.24 m/s to a post-assessment mean of 1.17 ± 0.29 m/s (*p* < 0.01) (Table 2; Figure 1).

### 3.4 Spatial parameters

**Figure 2:**
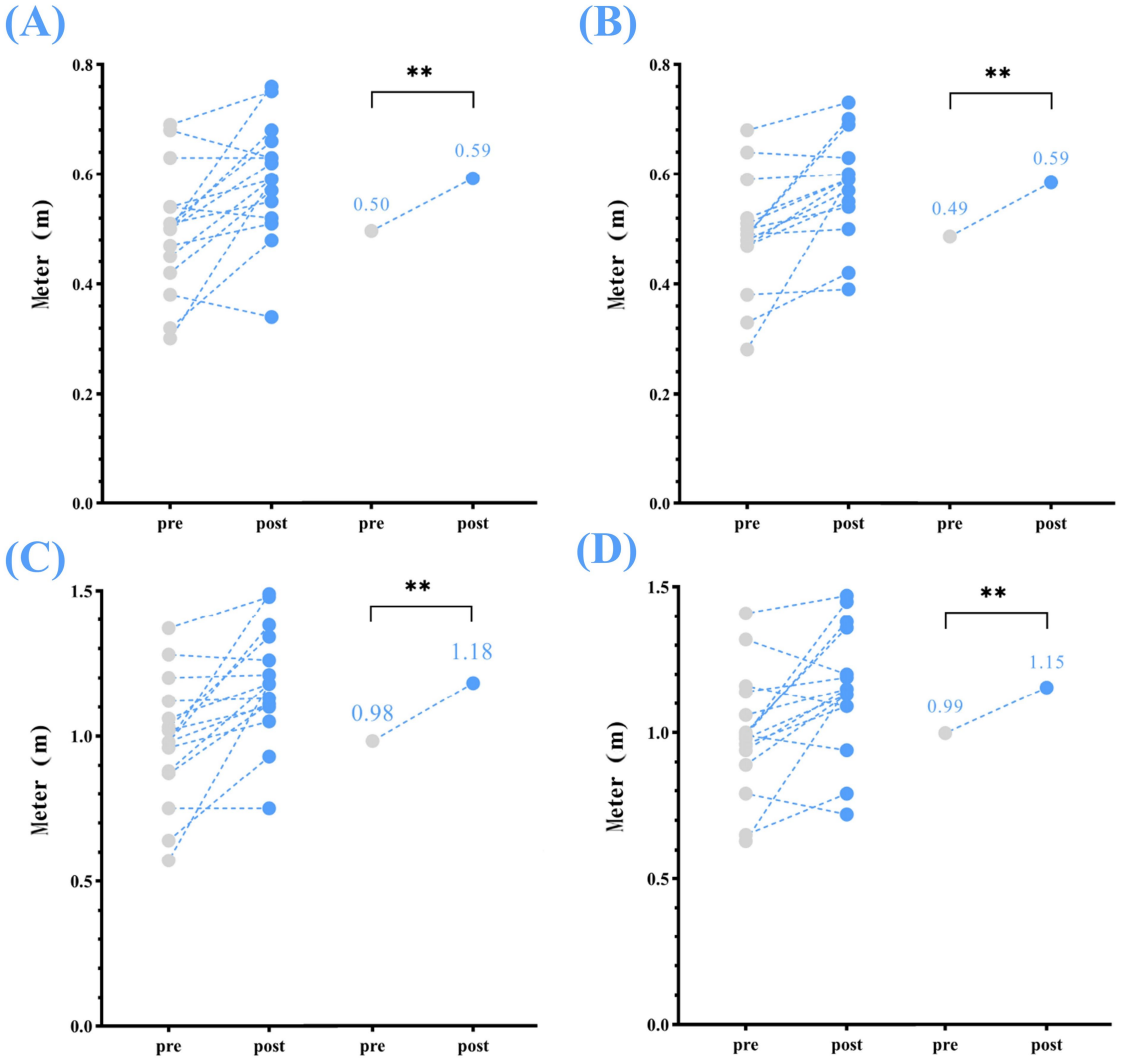
Spatial parameters—changes in step and stride length (left and right) between pre- and post-home-based VT. The following abbreviations are used: **(A)** step length (left); **(B)** step length (right); **(C)** stride length (left); **(D)** stride length (right); The circles on the left represent individual participants; the circles on the right represent the group mean for the pre- and post-intervention assessments.

Significant increase were observed for all spatial parameters from pre- to post-home-based VT, including: step length (left) increased by 0.09 m, step length (right) by 0.10 m, stride length (left) by 0.20 m, and stride length (right) by 0.16 m (*p* < 0.01) (Table 2; Figure 2).

### 3.5 Temporal parameters

**Figure 3:**
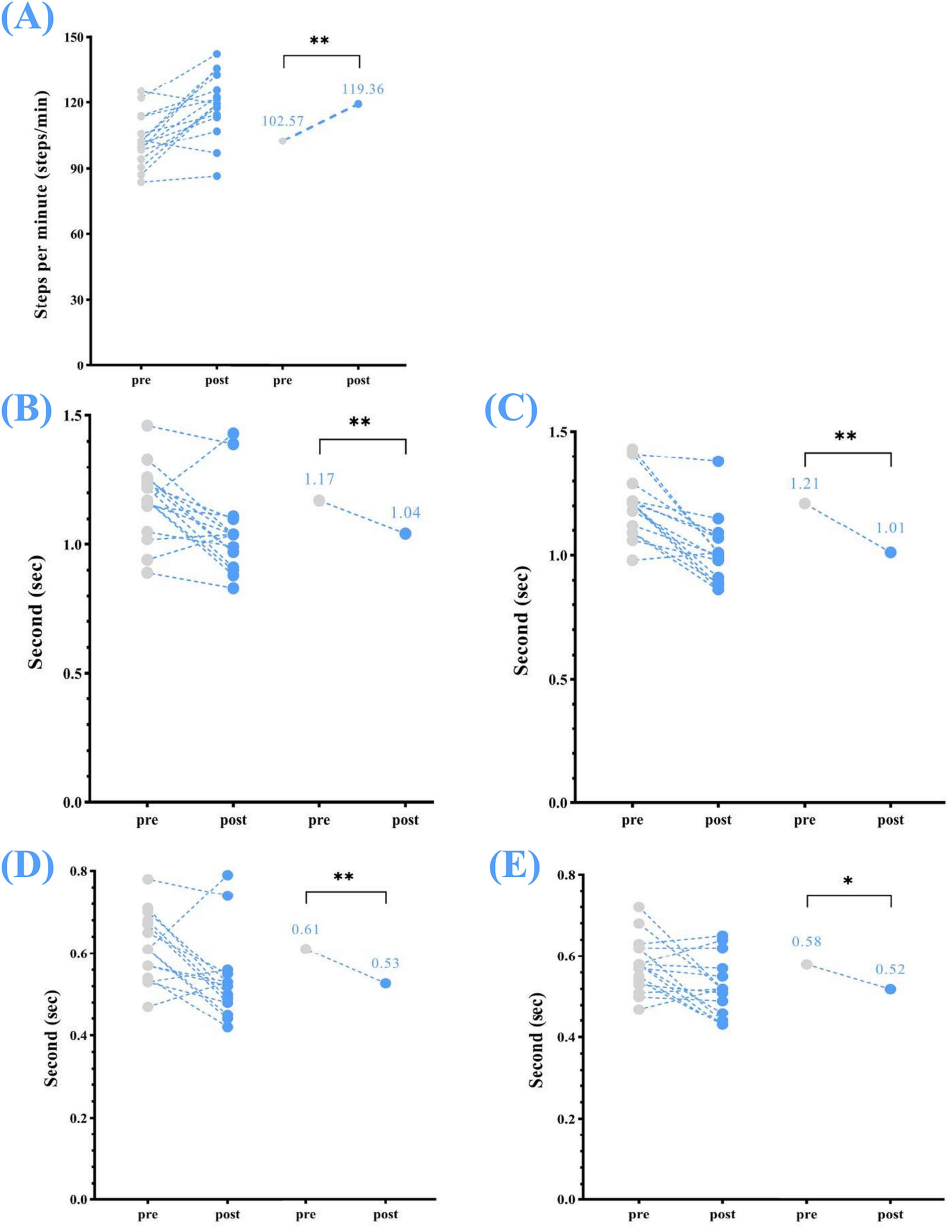
Temporal parameters—changes in step and stride times (left and right) and cadence between pre- and post-home-based VT. The following abbreviations are used: **(A)** cadence; **(B)** stride time (left); **(C)** stride time (right); **(D)** step time (left); **(E)** step time (right). The circles on the left represent the individual participants; the circles on the right represent the group mean for pre- and post-intervention. Home-based VT program.

Changes were also noted in temporal parameters, including: stride time (left) reduced from a mean (s) of 1.17 to 1.04, *p* < 0.01; stride time (right) reduced from 1.21 to 1.01, *p* < 0.01; step time (left) reduced from 0.61 to 0.53, *p* < 0.01; step time (right) reduced from 0.58 to 0.52, *p* < 0.05; and cadence increased from 102.57 steps/min to 119.36 steps/min, *p* < 0.01 (Table 2; Figure 3).

### 3.6 Temporophasic parameters

**Figure 4:**
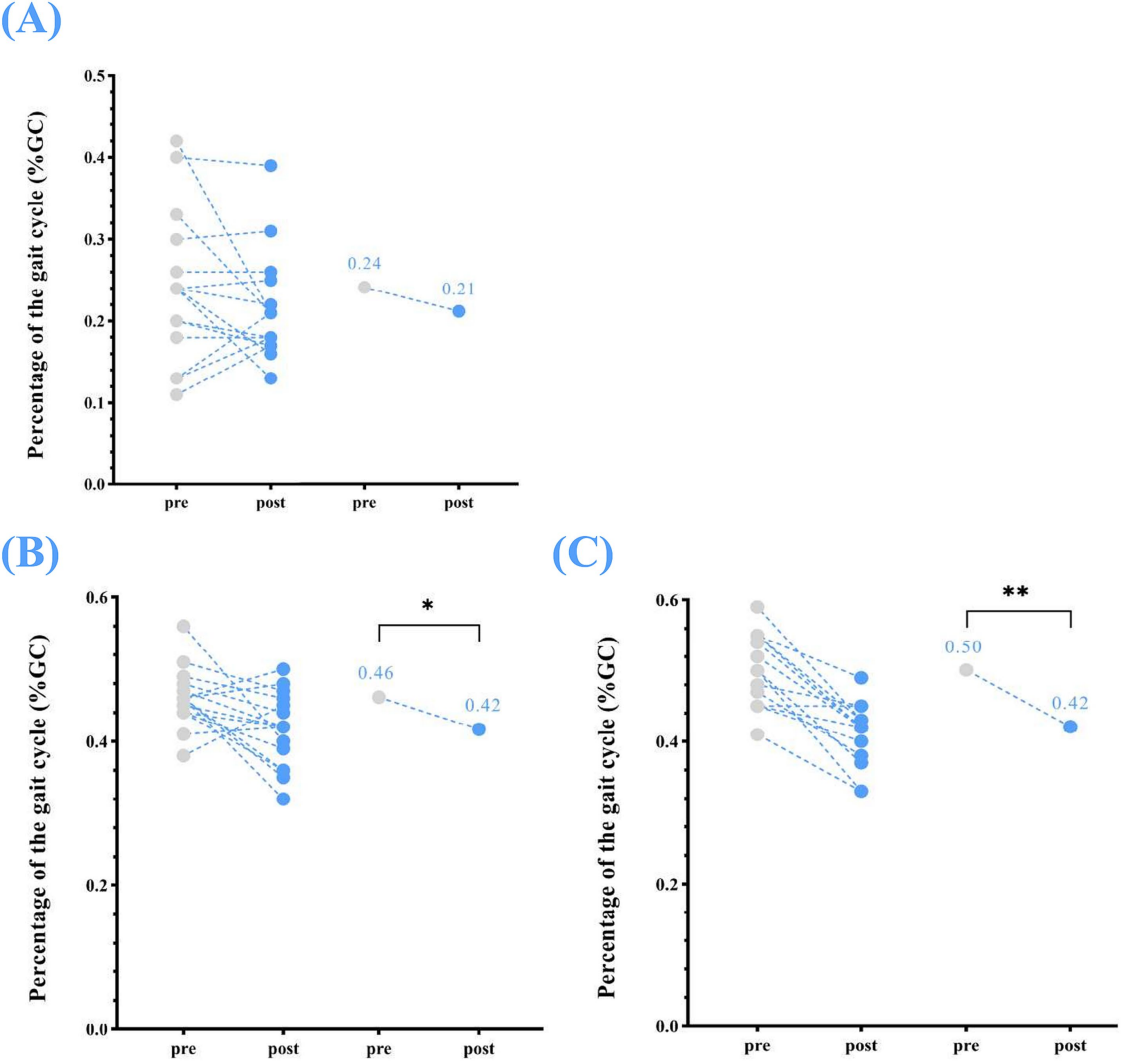
Temporophasic parameters—changes in single support (left and right) and double support between pre- and post-home-based VT. The following abbreviations are used: **(A)** double support **(B)** single support (left); **(C)** single support (right). The circles on the left represent individual participants; the circles on the right represent the group mean of pre- and post-intervention.

Significant changes were also observed in single support (left), which reduced from 0.46 ± 0.04% GC at pre-assessment to 0.42 ± 0.05% GC at post-assessment (*p* < 0.05). Similarly, single support (right) from a median (IQR) of 0.50 (0.50, 0.50)% GC at pre-assessment to 0.42 (0.40, 0.40)% GC and at post-assessment (*p* < 0.01). While reduced in double support were noted after the home-based VT program, the changes were not statistically significant (double support: 0.24 ± 0.09% GC to 0.21 ± 0.07% GC, *p* = 0.392) (Table 2; Figure 4).

### 3.7 Feasibility

All participants completed the home-based VT program with 100% adherence, and no adverse events were reported.

## 4 Discussion

Gait impairments in individuals with DS involve spatiotemporal gait parameters, angular changes in limb joints, and increased gait variability (48–50). Studies have shown that reduced gait parameters, such as walking speed and stride length, are often caused by ligament laxity and decreased muscle tone, leading to gait instability (51). As a result, individuals with DS tend to adopt a more cautious walking style, further reducing their stride length and gait speed. Since gait is fundamental to daily activities, normal gait development is crucial for overall healthy development. Unfortunately, from early childhood, the gait of individuals with DS is markedly different from that of typically developing individuals, leading to motor deficits that limit daily activities and discourage physical activity (15, 52). Studies of people with intellectual disabilities have shown lower levels of physical activity throughout their lives (53). Additionally, while gait typically improves as typically developing children grow, the gait of children with DS often remains impaired, emphasizing the need for sustained rehabilitation to address these deficits (10).

To our knowledge, this is the first study to explore the feasibility and effects of home-based VT in individuals with DS. Our findings show significant improvements in spatiotemporal gait parameters, including walking speed, stride time, step time, cadence, step length, stride length and single support following a two-week home-based VT program. However, changes in the gait cycle (double support) were not statistically significant. Importantly, no adverse events occurred, and all participants completed the program, demonstrating excellent adherence. These results align with previous studies that have reported improvements in gait function following VT (31). However, the existing research on VT primarily focuses on healthy adults, children, and individuals with neurological disorders such as multiple sclerosis, stroke, or cerebral palsy. Research on the use of VT in individuals with DS is extremely limited, highlighting the unique contribution of our study in this area.

One of the most critical gait indicators in our study was walking speed. There is substantial evidence supporting the importance of walking speed across the life span, as it is closely related to quality of life and overall mortality, earning it the designation of the “sixth vital sign” (54). Previous studies have demonstrated that reduced gait speed in individuals with DS leads to compensatory movement patterns during walking (48), further underscoring the need to improve gait speed to reduce these compensations (10, 55, 56). While other interventions like treadmill training, body-weight-supported gait training, and telehealth exercise programs have shown improvements in gait speed for children with DS (57), there has been a paucity of evidence on the effects of VT on spatiotemporal gait parameters in this population—until now. In our study, walking speed improved significantly (by 0.23 m/s) after the VT program, with effect sizes indicating moderate improvements. VT impact on walking speed may be explained by enhanced automatic postural reactions and increased muscle activation resulting from precisely guided reflex-based movements.

Although our findings indicate a significant improvement in walking speed, further study is required to determine if these results have clinical significance. For example, studies on stroke patients have suggested that improvements of 0.175 m/s or more in gait speed and increases of 0.08–0.14 m/s in older adults are considered clinically meaningful (58, 59). Thus, while our results are promising, larger studies are needed to confirm whether the improvements observed in individuals with DS meet the threshold for clinical significance.

In addition to walking speed, other spatiotemporal gait parameters—such as cadence, step length, stride length, stride time, and step time—also improved after VT. These improvements likely contributed to the overall enhancement in walking speed. For instance, previous research has shown that stride length is closely related to dynamic balance during walking (60). Individuals with DS often have shorter stride lengths due to poor dynamic balance, so improving stride length is crucial for enhancing gait quality (10). In our study, the increase in stride length following VT suggests that therapy not only improved walking speed but also enhanced overall gait quality. However, while improvements in the gait cycle (single and double support) were observed, they did not reach statistical significance. This may be due to the limited sample size, which limits the statistical power of the study.

Our study contributes to the limited evidence supporting the feasibility of home-based physiotherapy, particularly VT. The fact that all participants successfully completed the program without any adverse events suggests that home-based VT is a promising approach to improving gait function in individuals with DS. Given the accessibility of VT, incorporating it into early rehabilitation programs could improve positive effects. Future research should focus on longer intervention durations, comparative effectiveness studies, and personalized VT adjustments to maximize its clinical benefits.

## 5 Limitations and strengths

While the sample size of this study was relatively small, it represents a crucial first step in evaluating home-based VT as a potential physical therapy method for improving gait function in individuals with DS. Our results demonstrated the feasibility of home-based VT and provide preliminary evidence that this therapy can be successfully implemented. These results will guide further research, particularly larger studies with longer follow-up periods, to further assess the impact of home-based VT on gait function.

However, several limitations must be acknowledged. First, the limited sample size and lack of a control group limit the generalizability of the findings and make it difficult to exclude potential confounding factors. Second, there may be age and gender differences in gait function within the sample, but sub-analysis could not be performed due to the limited number of participants. Future studies with larger sample sizes are needed to consider these factors and provide more reliable conclusions. Third, assessing gait performance 48 h after the intervention minimizes the immediate activation effect but may not fully capture the progression of VT-induced neuromuscular adaptations. Future studies should include multiple follow-up assessments at different time points, including short-term (e.g., 12, and 24 h after the intervention) and long-term (e.g., 1 week, 4 weeks after the intervention), to more fully understand the temporal dynamics and sustainability of the VT effect. Fourth, The high adherence rate observed may be partially attributed to the short two-week intervention. However, whether this adherence will persist in long-term programs remains uncertain. Future research should examine long-term adherence to assess the long-term feasibility of home VT. Last, while this study focused on spatiotemporal gait parameters, incorporation of kinematic assessments in future research will provide deeper insights into joint coordination, neuromuscular adaptations, and the biomechanical mechanisms underlying VT effects.

## 6 Conclusion

This first prospective study provides promising evidence that a 2-week home-based VT program can improve gait ability in persons with DS. While the results offer valuable preliminary insights into the potential benefits of home-based VT, the small sample size means these findings should be interpreted cautiously. Future research with larger sample sizes, longer treatment periods, and more robust designs can be essential to fully understanding the impact of home-based VT on gait ability in individuals with DS.

## Data Availability

The original contributions presented in the study are included in the article/Supplementary material, further inquiries can be directed to the corresponding authors.
