# Supplementary figures and images for: Exploring the impact of home-based Vojta therapy on gait performance in individuals with Down syndrome: a preliminary feasibility study
Source: Front Neurol. 2025 Mar 11;16:1537635. doi: 10.3389/fneur.2025.1537635 (PMC11932913; doi:10.3389/fneur.2025.1537635)

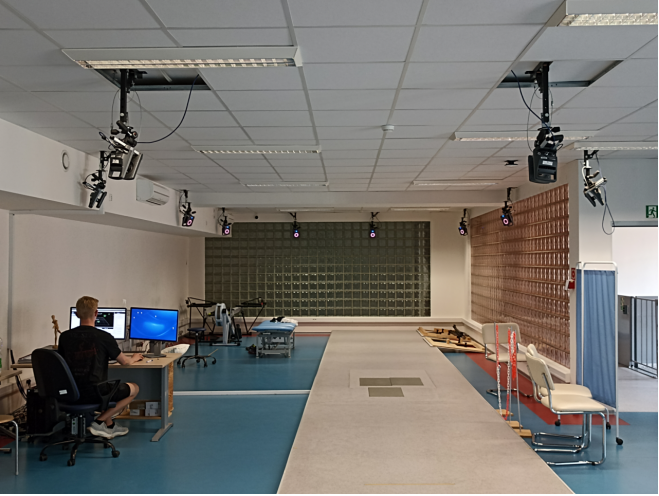

Supplement: Supplementary file 1 [file Image_1.JPEG]

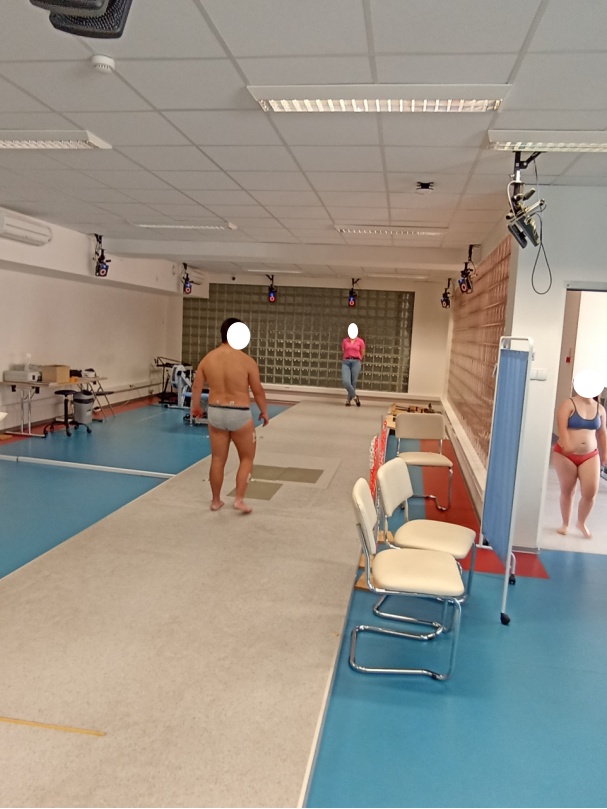

Supplement: Supplementary file 2 [file Image_2.JPEG]

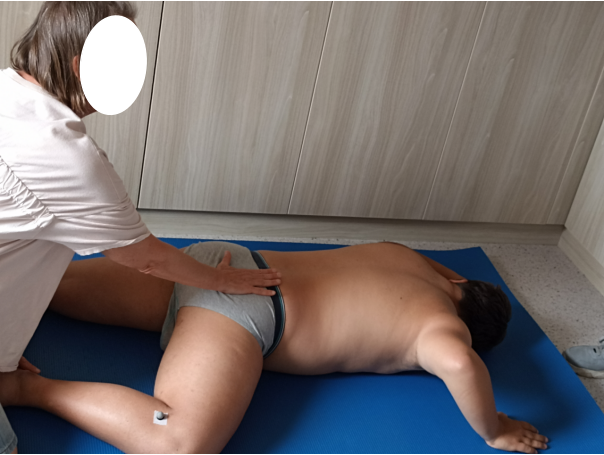

Supplement: Supplementary file 3 [file Image_3.JPEG]

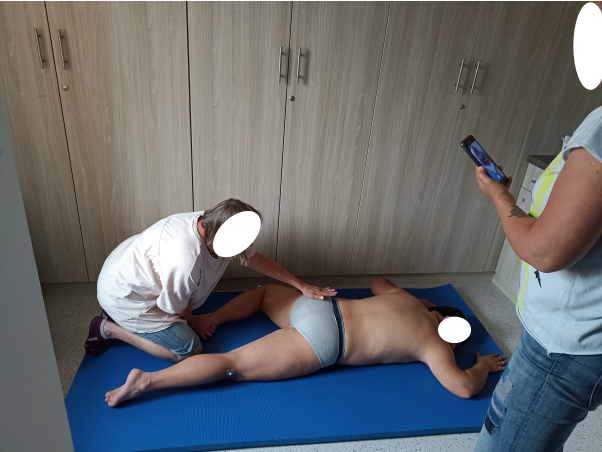

Supplement: Supplementary file 4 [file Image_4.JPEG]

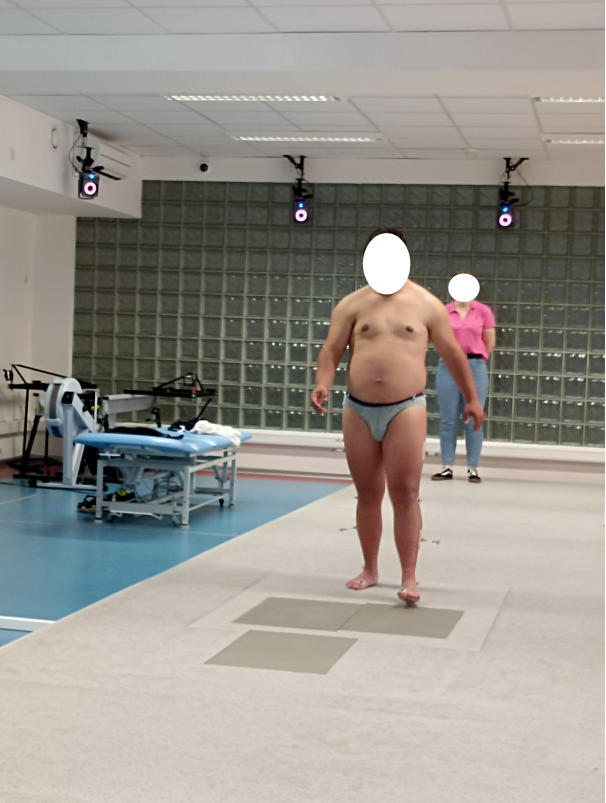

Supplement: Supplementary file 5 [file Image_5.JPEG]

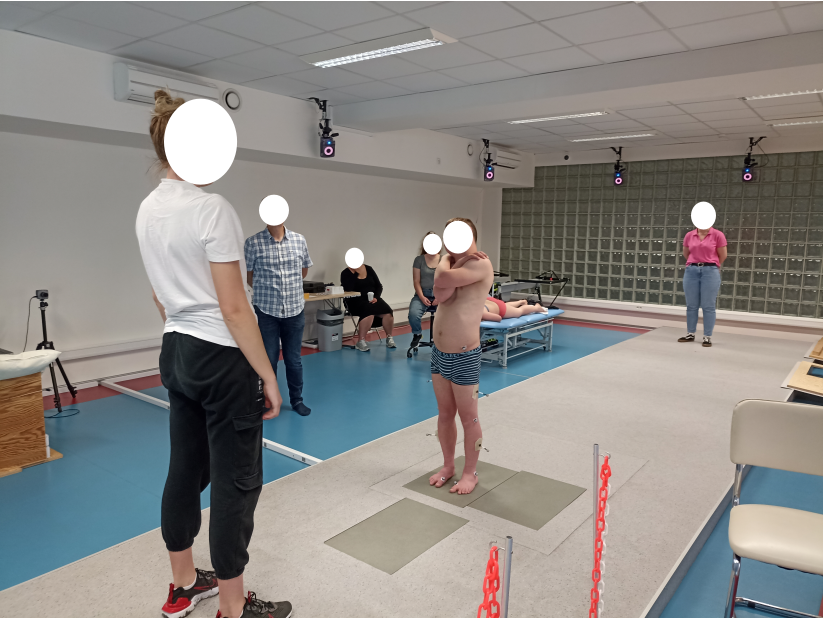

Supplement: Supplementary file 6 [file Image_6.JPEG]
