# Supplementary material for: Exploring the impact of home-based Vojta therapy on gait performance in individuals with Down syndrome: a preliminary feasibility study
Source: Front Neurol. 2025 Mar 11;16:1537635. doi: 10.3389/fneur.2025.1537635 (PMC11932913; doi:10.3389/fneur.2025.1537635)
Supplement: Supplementary file 7 [file Table_1.docx]

Supplementary Material

**Supplementary Table 1.** Characteristics of the participants (n = 16).

| Samples | Gender | Age (year) | Height (m) | Weight (kg) | Body Mass Index (kg/m2) |
| --- | --- | --- | --- | --- | --- |
| P1 | F | 15 | 1.68 | 65.10 | 23.07 |
| P2 | F | 14 | 1.41 | 65.00 | 32.69 |
| P3 | F | 12 | 1.55 | 49.90 | 20.77 |
| P4 | M | 17 | 1.85 | 63.10 | 18.44 |
| P5 | M | 23 | 1.60 | 54.60 | 21.33 |
| P6 | M | 17 | 1.63 | 77.00 | 28.98 |
| P7 | M | 18 | 1.54 | 42.20 | 17.79 |
| P8 | F | 15 | 1.40 | 57.60 | 29.39 |
| P9 | M | 17 | 1.56 | 55.20 | 22.68 |
| P10 | F | 19 | 1.58 | 80.50 | 32.45 |
| P11 | F | 23 | 1.59 | 69.10 | 27.51 |
| P12 | F | 30 | 1.67 | 78.00 | 27.97 |
| P13 | M | 19 | 1.64 | 65.80 | 24.46 |
| P14 | M | 14 | 1.61 | 59.40 | 22.92 |
| P15 | F | 13 | 1.42 | 48.40 | 24.00 |
| P16 | M | 20 | 1.48 | 51.70 | 23.60 |

Note: Table stand abbreviations include F: female; M: male; m: meter and kg: kilograms.
